# Renal-protective effects of Chinese medicinal herbs and compounds for diabetic kidney disease in animal models: protocol for systematic review and meta-analysis

**DOI:** 10.1186/s13643-023-02446-4

**Published:** 2024-01-12

**Authors:** Meifang Liu, Yuan Ming Di, Anthony Lin Zhang, Junhui Chen, Ruobing Wang, Juan Huang, Lei Zhang, Charlie Changli Xue, Xusheng Liu

**Affiliations:** 1 The China-Australia International Research Centre for Chinese Medicine, School of Health and Biomedical Sciences, RMIT University, Melbourne, Australia; 2 State Key Laboratory of Dampness Syndrome of Chinese Medicine, The Second Affiliated Hospital of Guangzhou University of Chinese Medicine, Guangzhou, China; 3 Department of Nephrology, Guangdong Provincial Hospital of Chinese Medicine, The Second Affiliated Hospital of Guangzhou University of Chinese Medicine, Guangzhou, China; 4 The Second Clinical College of Guangzhou University of Chinese Medicine, The Second Affiliated Hospital of Guangzhou University of Chinese Medicine, Guangzhou, China; 5 Pharmaceutical Research Department for New Drug Development and Authentication of Chinese Medicines, Guangdong Provincial Hospital of Chinese Medicine, The Second Affiliated Hospital of Guangzhou University of Chinese Medicine, Guangzhou, China

## Abstract

**Background:**

Diabetic kidney disease (DKD) is a common and severe complication of diabetes that can lead to end-stage renal disease with no cure. The first-line drugs recommended by clinical guidelines fail to achieve satisfactory effects for people with DKD. A Chinese herbal medicine Tangshen Qushi Formula (TQF) shows preliminary efficacy and safety in preserving renal function for people with DKD, but the effects on comprehensive renal outcomes remain unclear. We will conduct a systematic review and meta-analysis to evaluate the effects of TQF herbs and their compounds identified from ultra-high performance liquid chromatography-MS/MS in diabetic animal models with renal outcomes.

**Methods:**

This protocol complies with the guideline Preferred Reporting Items for Systematic Review and Meta-Analysis Protocols. We will include studies investigating the effects of TQF herbs and compounds on diabetic rats or mice with renal outcomes. Six electronic databases will be searched from their inception to February 2023. Quality assessment will be conducted using SYRCLE’s risk of bias tool. Standardized or weighted mean differences will be estimated for renal outcomes (creatinine, urea, proteinuria, histological changes, oxidative stress, inflammation, and kidney fibrosis). Data will be pooled using random-effects models. Heterogeneity across studies will be expressed as *I*^2^. Sensitivity analyses will explore treatment effects in adjusted models and within subgroups. Funnel plots and Egger’s test will be used to explore publication bias.

**Discussion:**

The results of this review will provide valuable insights into the potential effects of TQF in managing DKD. The limitation is that the included studies will be animal studies from specific databases, and the interpretation of the findings must be cautious.

**Systematic review registration:**

PROSPERO CRD42023432895. Registered on 19 July 2023 (https://www.crd.york.ac.uk/PROSPERO/#recordDetails).

**Supplementary Information:**

The online version contains supplementary material available at 10.1186/s13643-023-02446-4.

## Introduction

Diabetic kidney disease (DKD) is defined as chronic kidney disease induced by diabetes characterized by increased urinary albumin excretion and progressive reduction in renal function [1]. DKD occurs in 20 to 40% of all diabetes [2] and accounts for the leading cause of end-stage kidney disease [3]. People with DKD are at very high risk of kidney failure, heart attack, stroke, and death, which causes a significant socio-economic burden worldwide [4]. DKD is initiated by metabolic network alterations of diabetes (hyperglycemia and dyslipidemia), which promote hemodynamic pathways, metabolic changes, inflammation, and fibrosis [5–8]. The clinical practice guideline for DKD recommends a multifaceted and interdisciplinary approach targeting multifactorial pathogenesis, including lifestyle management, drug therapies, and risk factor control of glycemia and blood pressure [9]. The first-line drugs such as angiotensin-converting enzyme inhibitors, angiotensin II receptor blockers, and sodium-glucose cotransporter-2 inhibitors showed efficacy in reducing the risk of composite renal endpoints compared with placebos [10–13]. However, the first-line drugs could not achieve satisfactory effects in delaying the DKD progression and have undesirable side effects [14, 15]. Complementary and adjuvant therapies show the potential to address the clinical gap.

Chinese herbal medicine (CHM) has been evaluated in treating DKD and demonstrated potential benefits and safety in improving kidney function [16–20]. The unique combination of multiple herbs in CHM formulations is believed to contribute to its therapeutic effects by targeting multiple pathophysiological pathways involved in DKD progression [20, 21]. *Tangshen Qushi Formula* (TQF) is a seven-herbal CHM for people with DKD: *Astragalus membranaceus* (Fisch.) *Bge. var. mongholicus* (Bge.) Hsiao [*huang qi*; Astragali mongholici radix], *Cuscuta australis* R.Br. [*tu si zi*; Cuscutae semen], *Prunus davidiana* (Carrière) Franch. [*tao ren*; Persicae semen], *Atractylodes chinensis* (DC.) Koidz. [*cang zhu*; Atractylodis lanceae rhizoma], *Citrus reticulata* Blanco [*chen pi*; Aurantii amari epicarpium et mesocarpium], *Centella asiatica* (L.) Urb. [*ji xue cao*; Centellae asiaticae herba], *Isaria cicadae* Miquel [*chan hua*; Isaria cicadae Miquel]. An exploratory single-arm clinical trial involving 79 participants reported that TQF could delay the decline of renal function and improve glucolipid metabolism markers for people with stage 2–4 DKD [22]. No severe adverse events were reported. The findings from this clinical study implicated that TQF may delay the progression of DKD with a sound safety profile. However, the comprehensive effects on renal outcomes and the potential mechanisms of TQF herbs in treating DKD remain unclear.

The active compounds of TQF may play a role in synergistic interactions and multiple target points in treating DKD. Identifying the constituent compounds is the essential step to uncovering the effects and possible mechanisms of TQF. Thus, we have conducted a chemical analysis of TQF complying with the standards established in the “Consensus Statement: The Phytochemical Characterization of Medicinal Plant Extracts” [23] and identified 38 active compounds via ultra-high performance liquid chromatography-mass spectrometry/mass spectrometry (UHPLC-MS/MS) (Additional file 1). To the best of our knowledge, no clinical trials specifically address TQF. The lack of available clinical trials on TQF drives our focus on preclinical studies. The preliminary search showed that sufficient animal studies on individual TQF herbs and compounds are available. Herein, we conduct a systematic review and meta-analysis to (1) evaluate the effects of TQF herbs and compounds on renal outcomes in animal studies, (2) identify the treatment-related factors (intervention type, dosage, treatment duration, and administration route) influencing TQF in DKD animal models, and (3) provide a comprehensive and unbiased synthesis of the available preclinical evidence on TQF for DKD.

## Methods

We design this systematic review and meta-analysis in line with the *Preferred Reporting Items for Systematic Review and Meta-Analysis Protocols (PRISMA-P) 2015* (Additional file 2) [24]. The methodology is guided by the Systematic Review Centre for Laboratory Animal Experimentation (SYRCLE) [25].

### Eligibility criteria

#### Study design

We will include controlled studies with two or more treatment groups. Studies that lack diabetes control groups or have a diabetes control group but do not have kidney impairment will be excluded. Conference abstracts, reviews, systematic reviews, meta-analyses, and other non-original studies will be excluded but will be used to search for additional articles.

#### Animals

The animals being studied by the review will be rats or mice with diabetes (either type I or type II diabetes) and kidney impairment, regardless of strains, sex, age, body weight, and modeling methods. Human studies and in vitro studies will be excluded.

#### Interventions

Studies using any TQF herb or compound as an intervention will be eligible, regardless of preparation method, administration route, dosage, and treatment duration. Studies compassing the combined therapy of TQF herbs or compounds as the intervention will be excluded. The studied compounds will be checked with the exact molecular formula according to the chemical profile.

#### Comparators

Included studies must have two or more groups, at least one control group of diabetes animals without the intervention (sham/vehicle) or with a different intervention to TQF herbs or compounds. Studies that lack appropriate diabetes control groups with kidney impairment will be excluded.

#### Outcomes

Studies that report renal outcomes with renal function and injury markers will be included. Renal function measures include serum creatinine (SCr), urea, or blood urea nitrogen (BUN). The injury markers could be urinary protein/albumin, kidney fibrosis, glomerulosclerosis, kidney size/weight, inflammation, and oxidative stress. Other outcomes include blood pressure, blood glucose, HbA1c, and lipids. All outcomes except for the listed ones in the inclusion criteria will be excluded. Only studies with complete, correct, or original data will be included.

#### Time points

We will include studies that have at least one time point after receiving the intervention. There are no restrictions on the treatment duration or length of the interval between baseline and follow-up.

#### Other selection criteria

Language is limited to English and Chinese.

### Databases and sources

We will search journal articles and theses in six electronic English- and Chinese-language-based databases from inception to February 2023, including PubMed, EMBASE, China Biomedical Literature, China National Knowledge Infrastructure, Chongqing VIP, and Wanfang.

### Search strategy

Before initiating this protocol, we conducted a literature search and did not identify any existing systematic or scoping reviews on the topic of renal-protective effects of TQF and its compounds for DKD animal models. Preliminary searches were done in January 2023 to identify the most relevant Mesh terms and keywords for our search. Combinations of the search terms were also tested so that we could identify the most relevant studies in our comprehensive search. A comprehensive search strategy has been designed to retrieve all potentially relevant animal studies adapted from Leenaars et al. [26]: (1) identify relevant search terms for TQF interventions (herbs and compounds), (2) identify relevant search terms for DKD, (3) identify relevant search terms for animal studies, and (4) search, combine and evaluate search results. The detailed search strategy in PubMed is provided in Table 1.

**Table 1:** A comprehensive search strategy to identify potentially relevant animal studies on TQF interventions

Strategy		Step	Details
(1)	Formulate research question	Formulate a focused research question consisting of the following: 1) Intervention/exposure 2) Disease of interest/health problem 3) Animal/animal species/population studied 4) Outcome measures	What are the effects of 1) TQF herbs and compounds on 4) renal outcomes in 3) rats or mice for 2) diabetic kidney disease?
(2)	Identify appropriate databases and sources of studies	• Identify both general biomedical and topic-specific databases • Select all relevant databases • Check other sources, such as reference lists	PubMed, EMBASE, China Biomedical Literature, China National Knowledge Infrastructure, Chongqing VIP, and Wanfang
(3)	Transform research question into search strategy	• Design and run a search strategy customized for each database • Start with a database that includes a thesaurus, e.g., PubMed or EMBASE • Involve an information specialist • Save citations (titles/abstract) in reference software • Document the applied search strategies	See Additional file 3 for details on the PubMed search strategy
(4)	Collect search results and remove duplicates	Combine saved citations of all databases into one file in the EndNote Library and remove citations that appear more than once	PubMed, *n* = 3942; EMBASE, *n* = 685; Chinese-language-based databases, *n* = 5173 Removing duplications (*n* = 5001)
(5)	Identify potentially relevant papers	Screen the title and abstract of the references and identify papers based on the potential relevance	Screen *n* = 4799

TQF, *Tangshen Qushi Formula*

We will identify the search terms for the TQF interventions (herbs and compounds) from the online scientific resources, Royal Botanic Gardens of Kew [27], Plants of the World [28], and the open chemistry database managed by the National Institutes of Health [29]. An example of the search strategy in PubMed is provided in a supplementary file (Additional file 3). Search history and records will be saved and documented. All the saved records will be transferred into EndNote 20 software. The duplicated citations will be compared and removed in the EndNote Library, and the record with more information will be kept while the others are deleted. De-duplication will be conducted for the second round in Microsoft Excel by finding, marking, and removing duplicates.

### Study selection

**Fig. 1:**
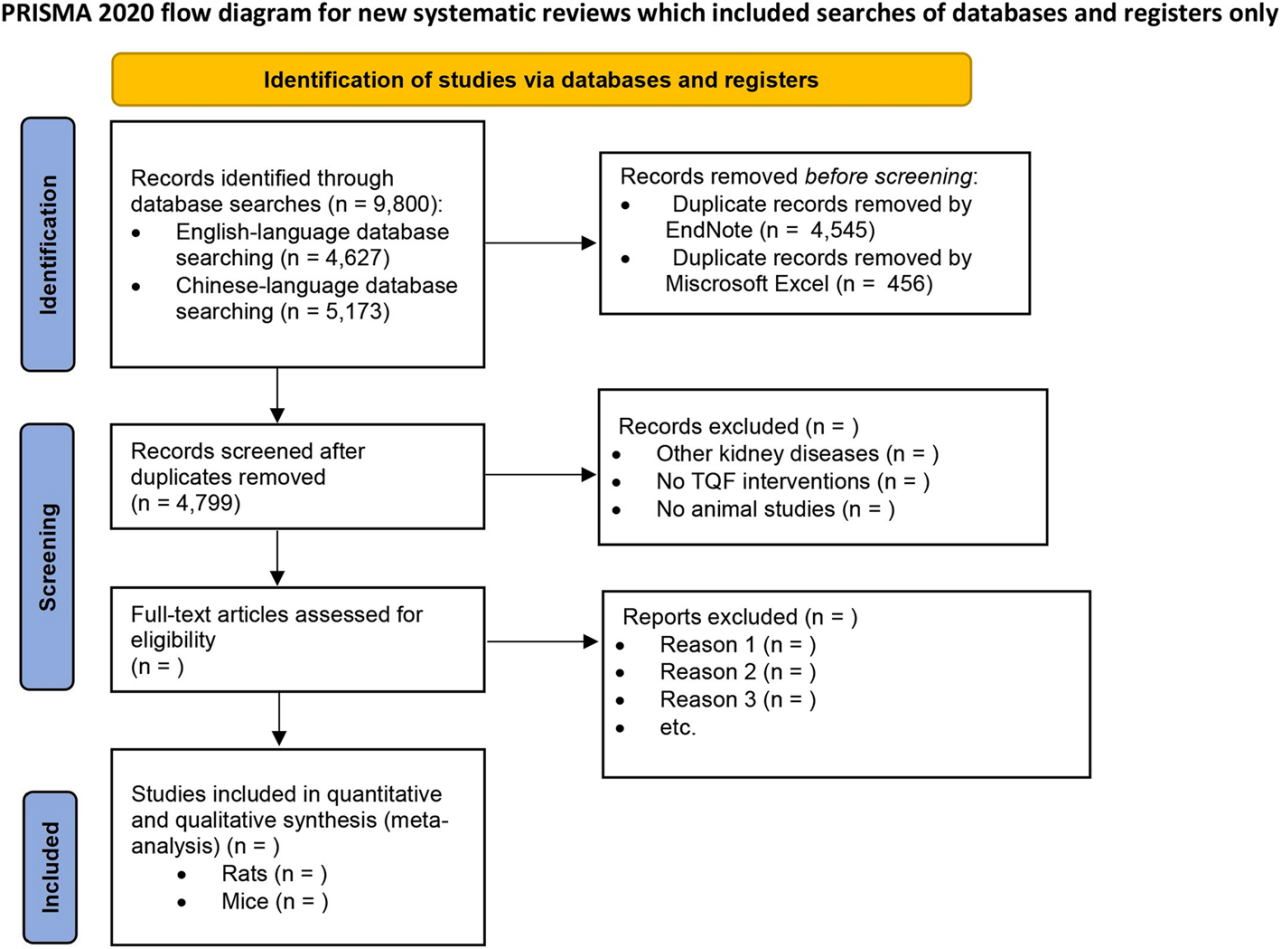
PRISMA flow diagram of study searching and selection. TQF, *Tangshen Qushi Formula*

Two screening phases will be performed. Titles and abstracts will be initially screened before full-text review. Two reviewers will screen titles and abstracts based on their relevance (ML, JC). The exclusion criteria are prioritized to avoid discrepancies between reviewers in the reason for exclusion recorded: (1) not an animal study, (2) not a diabetic animal model, (3) lack of diabetes control group, (4) not TQF herbs or compounds as the intervention, and (5) without reporting renal outcomes. Full-text reviews will be conducted in two rounds, with the first (ML, RW) round to assess the inclusion and exclusion criteria and the second (ML, JC) round for double check. If there are discrepancies, a third review will be performed (YD). A PRISMA flow diagram of study searching and selection will be provided (Fig. 1).

### Data extraction

Two reviewers (ML and RW) will conduct data extraction independently. Data will be collected from the main text, tables, and figures of eligible studies using a pre-designed Microsoft Excel template (Additional file 4). All outcomes will be extracted in original numbers with actual units. Results in figures will be captured using the WebPlotDigitizer online tool. Missing data will be requested via email to the corresponding author; if there is no reply from them within 14 days, the data will be treated as incomplete and will not be used in the analysis.

The data will be extracted by the following components:

- Study information: authors, publication year, article title, journal, and country
- Study design: groups (healthy control group, diabetic control group, intervention group, and/or positive drug control group), randomization, and/or blinding methods
- Animal demographic: species, strain, sex, age, weight, and induction of diabetes
- Intervention protocol: intervention measures, sample size, final number in analysis, intervention measure, administration route, dosage, and treatment duration
- Renal outcomes: SCr, urea or BUN, urinary protein/albumin excretion rate (UPER/UAER), urinary protein/albumin-to-creatinine ratio (UPCR/UACR), blood pressure (BP), blood glucose (BG), HbA1c, cholesterol, triglyceride (TG) and kidney size, kidney injury markers with actual units (including glomerular size, mesangial changes, markers of inflammation, fibrosis, and oxidative stress)—all continuous data, presented as end-of-test mean value and standard deviation (SD)/standard error of the mean (SEM).

SEM will be converted to SD: SD = √*n* × SEM (*n* means the number of analyses) when needed. The units will be converted consistently across studies whenever possible.

### Quality assessment and risk of bias assessment

The reporting quality of included studies will be assessed using a scoring system with 10 domains adapted from Hickson et al. [30], clear definition or presentation of each domain will score one point, higher the total score indicates higher reporting quality. The ten domains are as follows: (1) animal characteristics (strain, age, sex), (2) number of animals per group, (3) animal model, (4) animal model validation, (5) AM preparation, (6) dosages, (7) administration route, (8) timing of intervention (relative to disease), (9) outcome measures, and (10) random allocation or stratification of animals to groups.

The methodological quality of included studies will be evaluated using the SYRCLE’s risk of bias tool [25]. This risk of bias tool is adapted from the Cochrane risk of bias (RoB) tool. SYRCLE’s RoB for animal studies contains ten entries relating to selection bias, performance bias, detection bias, attrition bias, reporting bias, and other biases. For individual studies, each domain of the risk of bias tool will be graded as “yes,” “no,” and “unclear” (meaning the high, unclear, or low risk of bias, respectively) with justifications. A funnel plot will be produced for creatinine outcome to assess the potential for publication bias. Two independent reviewers (ML, YD) will perform the scoring of quality assessment and risk of bias with a third reviewer (LZ) for discrepancies.

### Data synthesis and analysis

The results from the experimental (TQF interventional animals) and control (diabetes animals) groups will be compared at the latest time point after the TQF-based intervention. Outcomes will be estimated as weighted mean difference (WMD). When different scales, units, or measurement methods are used for an outcome, standardized mean differences (SMD) will be used. Results will be presented in WMD or SMD with 95% confidence intervals for all the renal outcomes. Random-effects model will be used to conduct a meta-analysis since the actual effect size is assumed to vary across studies due to inherent differences in animal models, interventions, and/or methodologies. Heterogeneity among the included studies will be expressed as the I^2^ [31]. I^2^ > 50% suggests substantial heterogeneity. Heterogeneity will be explored by conducting subgroup analyses or meta-regression.

The meta-analysis will be performed for all outcome measures reported in at least two articles. If meta-analysis is not possible, data will be reported through a descriptive summary. Subgroup analyses of these outcomes will be considered to assess the animal- and treatment-related effects of TQF-based therapy. For animal-related effect analyses, species (rat, mouse), sex (male, female), and diabetes induction (streptozotocin, db/db, other) will be compared. Treatment-related effect analyses will include intervention type (herb or compounds), administration route (oral gavage, intraperitoneal injection, other routes), dosage (low, medium, high), and treatment duration (short-term, medium-term, and long-term). Differences between the groups will be evaluated using an interaction test, as suggested by Altman and Bland [32]. We will conduct sensitivity analysis through a leave-one-out method that excludes one or more studies of lower quality or one that appears to be an outlier. Publication bias will be assessed using funnel plots and Egger’s test [33]. Funnel plots will be performed if at least ten studies were available in the meta-analysis. The analysis will be conducted using Review Manager Software 5.3 and Stata 15.0.

This systematic review has been registered at the International Prospective Register of Systematic Reviews (ID: CRD42023432895).

## Discussion

Chinese medicinal herbs and compounds are a treasure trove for natural products. Growing evidence shows that Chinese medicinal herbs and compounds deliver renal protective effects by regulating the renin–angiotensin–aldosterone system, oxidative stress, inflammation, and apoptosis in DKD animal models [34–37]. Similarly, herbs and compounds from TQF show renal-protective effects via these identified pathways. Astragaloside IV alleviates renal tubular epithelial-mesenchymal transition via the CX3CL1-RAF/MEK/ERK signal pathway in DKD mice [38]. Calycosin regulates ferroptosis both in cell lines and mice [39]. Hesperetin presents renal benefits by activating Nrf2/ARE/glyoxalase 1 and TGFβ1- ILK-Akt, thus ameliorating DKD in animal models [40, 41]. Research advances show inflammation, fibrosis, and oxidative stress are the key pathways contributing to renal function impairment and albuminuria in the course of DKD [42]. The interactive relationship between oxidative stress and renal damage involves inflammation and fibrosis in DKD [43].

The planned analysis approach for the renal outcomes will provide the ability to synthesize the critical renal injury markers: SCr, BUN, albuminuria, histological changes, oxidative stress, fibrosis, and inflammation. The current systematic review protocol outlines a comprehensive approach to evaluate the interactions of TQF herbs and compounds with inflammation, fibrosis, and oxidative stress. The results on renal outcomes may implicate the possible mechanisms that affect the effects in DKD animal models. The controlled animal studies allow for a better investigation of TQF’s effects on renal outcomes in DKD models. By following a systematic methodology, this review protocol ensures that the search strategy, study selection criteria, and data extraction processes are rigorous and unbiased. The anticipated findings from this systematic review will shed light on the therapeutic effects of TQF-based interventions in ameliorating renal dysfunction and improving pathological processes associated with DKD.

Further, variations in study designs, animal models, intervention protocols, outcome measures, and quality of included studies can contribute to heterogeneity across the studies. This heterogeneity may limit the ability to draw conclusive findings, which should be appropriately managed. Additionally, animal models may not fully reflect the complexity of human DKD, and the translation of findings from animal studies to human clinical practice could be challenging. The interpretation of the findings should be cautious.

This will be the first systematic review of TQF herbs and compounds based on the chemical analysis results identified from the UPLC-MS/MS method. By synthesizing the available results from animal studies, this review will provide valuable insights into the potential benefits of TQF in managing DKD. Additionally, this review will provide estimates for the effects of animal- and treatment-related factors influencing TQF in DKD animal models. The results of this systematic review will contribute to a better understanding of the therapeutic potential of Chinese medicinal herbs and compounds in DKD and inform future preclinical and clinical investigations.

### Supplementary Information

**Additional file 1.** Preparation and chemical analysis of TQF.

**Additional file 2.** PRISMA-P 2015 checklist.

**Additional file 3.** Search strategy in PubMed example.

**Additional file 4.** Data extraction template.

## Data Availability

Not applicable.

## Abbreviations

DKD: Diabetic kidney disease
CHM: Chinese herbal medicine
TQF: Tangshen Qushi Formula
eGFR: Estimated glomerular filtration rate
HbA1c: Hemoglobin A1c
UPLC: Ultra-high performance liquid chromatography
MS: Mass spectrometry
PRISMA-P: Preferred Reporting Items for Systematic Review and Meta-Analysis Protocols
SYRCLE: Systematic Review Centre for Laboratory Animal Experimentation
BUN: Blood urea nitrogen
SD: Standard deviation
SEM: Standard error of the mean
UACR: Urinary albumin-to-creatinine ratio
UPCR: Urinary protein-to-creatinine ratio
RoB: Risk of bias
WMD: Weighted mean difference
SMD: Standardized mean difference
