# Supplementary material for: Renal-protective effects of Chinese medicinal herbs and compounds for diabetic kidney disease in animal models: protocol for systematic review and meta-analysis
Source: Syst Rev. 2024 Jan 12;13:23. doi: 10.1186/s13643-023-02446-4 (PMC10785383; doi:10.1186/s13643-023-02446-4)
Supplement: Supplementary file 1 — Additional file 1. Preparation and chemical analysis of TQF. [file 13643_2023_2446_MOESM1_ESM.pdf]

Preparation and chemical analysis of TQF

Preparation of TQF granules

TQF was prepared as an oral granulated product by the Jiangyin Tianjiang Pharmaceutical Company (Jiangyin, China), a Good Manufacturing Practice (GMP) certificated company. The amount of each botanical drug in the preparation of every kilogram of TQF granules was *Astragalus mongholicus Bunge*, *Cuscuta australis R.Br.*, *Prunus davidiana (Carrière) Franch.*, *Atractylodes lancea (Thunb.) DC.*, *Citrus × aurantium L*, *Centella asiatica (L.) Urb.*, *Isaria cicadae Miquel*. The raw plant materials were acquired from commercial sources. The scientific name, species name, pharmaceutical name, Chinese Pin Yin name, proportion, and place of origin were provided in the **S1 Table**. The purity/contaminant tests showed that the TQF granules were free of heavy metals or other undesirable contaminants, which complied with the pharmacopeial criteria (2020 Edition (34)). The stability/consistency tests are being performed to indicate whether the product is stable/consistent from the start till the end of the clinical study. No equivalence test was needed since a single batch of TQF granules would be used during the clinical study.

S1 Table The components of TQF in scientific, species, pharmaceutical, and Chinese Pin Yin names, proportion, and place of origin

**Scientific name**	**Species name**	**Pharmaceutical name**	**Chinese pin yin**	**Proportion**	**Place of origin (Commercial supplier/province)**
Astragalus mongholicus Bunge	*Fabaceae*	*Astragali mongholici radix*	*Huang qi*	20	Anhui Cunzhen Pharmaceutical Co., Ltd./Gansu
Cuscuta australis R.Br.	*Convolvulaceae*	*Cuscutae semen*	*Tu si zi*	15	Anhui Baiyutang Traditional Chinese Medicine Decoction

					Pipe Co., Ltd./Inner Mongolia
Prunus davidiana (Carrière) Franch.	*Rosaceae*	*Persicae semen*	*Tao ren*	10	Anhui Haixin Pharmaceutical Co., Ltd./Gansu
Atractylodes lancea (Thunb.) DC.	*Asteraceae*	*Atractylodis lanceae rhizoma*	*Cang zhu*	10	Anhui Fengliaoxing Pharmaceutical Co., Ltd./Inner Mongolia
Citrus × aurantium L.	*Rutaceae*	*Aurantii amari epicarpium et mesocarpium*	*Chen pi*	10	Anhui Haixin Pharmaceutical Co., Ltd./Zhejiang
Centella asiatica (L.) Urb.	*Apiaceae*	*Centellae asiaticae herba*	*Ji xue cao*	15	Anhui Haixin Pharmaceutical Co., Ltd./Anhui
Isaria cicadae Miquel	*Cordycipitaceae*	*Isaria cicadae Miquel*	*Chan hua*	8	Anhui Jinguoyuan Pharmaceutical Co., Ltd./Anhui

The extraction procedure of the TQF product was: firstly, do two rounds of decocting for the mixture of seven raw botanical drugs with water. Secondly, the decoctions from the previous step are merged, then filtered and concentrated into a transparent paste. Next, maltodextrin was added to the paste till it was dissolved and then spray-dried. The mixture was made into 1000 grams of granules. In the end, the granules were packaged into packets of 7.5 grams. A single batch was used for this clinical study.

Preparation of placebo

The TQF placebo was produced by the same manufacturer using a concentrate of caramel pigments, gardenia yellow pigment, sunset yellow pigment, and dextrin with the proportion of 1.5: 1: 1: 0.8. Other components were sucrose octaacetate (3%), suspending agent (dextrin and titanium dioxide, 0.8%), and lactose (10%). The placebo was made in granules and packaged into packets of 7.5 grams. According to the 2020 Chinese pharmacopeia, the components in the placebo are food-grade at this dosage and have no known side effects with oral consumption. The blinding test of the TQF granules and placebo showed the package

and weight were identical, and the appearance, color, smell, and taste were similar. Critical information on chemicals, manufacturing, and control of TQF and TQF placebo is being conducted by the pharmaceutical team of GPHCM. The chemical composition of TQF is validated using high-performance liquid chromatography/mass spectrometry.

Chemical analysis and quality control

Ultra-high performance liquid chromatography-mass spectrometry/mass spectrometry (UHPLC-MS/MS) analysis was adopted for the TQF granules. The supernatant of granules was ultrasonically extracted with 50% methanol. UHPLC analysis was performed using a Thermo UltiMate 3000 UHPLC instrument (Waltham, MA, USA). The analysis employed an ACQUITY UPLC® HSS T3 C18 column (2.1 mm × 100 mm, 1.8 µm, Waters, USA). The mobile phase comprised acetonitrile (A) and water with 0.1% formic acid (B). The flow rate was maintained at 200 µL/min. A gradient elution system was employed as follows:

Time (min)	% A
0-2	3
2-3	3-10
3-5	10
5-8	10-20
8-13	20
13-18	20-40
18-22	40
22-24	40-80

An injection volume of 5 µL was used for each sample.

Mass Spectrometry:

Mass spectrum acquisition was carried out using a Q-Exactive hybrid quadrupole-orbitrap mass spectrometer (Thermo Scientific, San Jose, USA) equipped with heated electrospray ionization (HESI) operating in the negative ionization mode. The main parameters optimized for mass spectrometry were set as follows:

Parameter	Setting
Spray voltage	-2.5 kV
Capillary temperature	350 °C
Auxiliary gas heater temperature	350 °C

Auxiliary gas flow rate	15
Sheath gas flow rate	40
Sweep gas flow rate	0

The raw data was imported into Compound Discoverer 3.0 software, and the possible molecular formulas were fitted through the extracted molecular ion chromatographic peaks. The results were then matched with the mz Vault and mz Cloud databases. Xcalibur 2.1 software was used to further analyze and identify the compounds by comparing them with relevant literature and standard substances based on the accurate m/z values of precursor and fragment ions, retention time, and the information of cleavage pattern.

Thirty-eight compounds were identified from UHPLC-MS/MS analysis of TQF (Fig 2 in the manuscript). The details of the compounds and their bioactivities are listed in the **S2 Table**.

S2 Table Compounds from UHPLC-MS/MS analysis of TQF and their bioactivities

No.	tR/min	[M+H]+	$[M-H]^-$ /$[M+FA-H]^-$	Molecular formula	Compounds	Predominant bioactivities
1	1.21	175.1188		$C_6H_{14}N_4O_2$	L (+)-Arginine *	Nitric oxide synthesis, vascular health, immune support
2	2.03		133.0126	$C_4H_6O_5$	Malic acid	Energy metabolism, Krebs cycle, cellular respiration
3	2.87	132.1018		$C_6H_{13}NO_2$	L-Norleucine *	Protein synthesis, enzyme activity
4	3.13	132.1018		$C_6H_{13}NO_2$	Isoleucine	Protein synthesis, immune function, energy metabolism
5	3.36	268.1045		$C_{10}H_{13}N_5O_4$	Adenosine	Energy transfer, cellular signaling, neurotransmission
6	3.80	284.0992		$C_{10}H_{13}N_5O_5$	Guanosine	RNA/DNA synthesis, neurotransmission
7	5.88	166.0861		$C_9H_{11}NO_2$	Alanine	Glucose metabolism, energy production
8	6.59		315.0719	$C_{13}H_{16}O_9$	Gentisoyl glucoside	Antioxidant, anti-inflammatory
9	7.79	355.1015	353.0880	$C_{16}H_{18}O_9$	Neochlorogenic acid	Antioxidant, anti-inflammatory

10	8.13	205.0967		$C_{11}H_{12}N_2O_2$	L-Tryptophan	Serotonin precursor, mood, sleep
11	12.97		609.1442	$C_{27}H_{30}O_{16}$	Rutin *	Antioxidant, anti-inflammatory, cardiovascular
12	9.77	355.1014	353.0882	$C_{16}H_{18}O_9$	Chlorogenic acid *	Antioxidant, glucose metabolism
13	10.01	355.1016	353.0874	$C_{16}H_{18}O_9$	Cryptochlorogenic acid	Antioxidant
14	10.14		502.1557	$C_{20}H_{27}NO_{11}$	Amygdalin *	Cyanide metabolism, potential anti-cancer
15	10.45	177.0545		$C_{10}H_8O_3$	hymecromone	Gallstone treatment
16	10.83	595.1650	593.1507	$C_{27}H_{30}O_{15}$	Vicenin -2	Antioxidant, potential anti-inflammatory
17	11.08		623.1605	$C_{28}H_{32}O_{16}$	stellarin-2	Limited information
18	11.64		367.1031	$C_{17}H_{20}O_9$	Feruloylquinic acid	Antioxidant, potential anti-inflammatory
19	12.70	565.1541	563.1395	$C_{26}H_{28}O_{14}$	vitexin- xyloside	Limited information
20	12.79		595.1664	$C_{27}H_{32}O_{15}$	Eriocitrin *	Antioxidant, potential cardiovascular
21	12.97	447.1275		$C_{22}H_{22}O_{10}$	Calycosin 7-O-glucoside *	Estrogenic, cardiovascular
22	13.09	595.1643	593.1508	$C_{27}H_{30}O_{15}$	Lonicerin	Limited information
23	13.51	465.1017	463.0878	$C_{21}H_{20}O_{12}$	Hyperoside *	Antioxidant, anti-inflammatory, cardiovascular
24	13.78	465.1021	463.0880	$C_{21}H_{20}O_{12}$	Isoquercitrin	Antioxidant, potential anti-inflammatory
25	14.99	517.1334	515.1189	$C_{25}H_{24}O_{12}$	Isochlorogenic acid B	Antioxidant
26	15.21	581.1849	579.1703	$C_{27}H_{32}O_{14}$	Narirutin	Antioxidant, potential anti-inflammatory
27	15.98	517.133	515.1191	$C_{25}H_{24}O_{12}$	Isochlorogenic acid A	Antioxidant

		1			*	
28	16.32		447.0919	$C_{21}H_{20}O_{11}$	Astragaline *	Immunomodulatory
29	17.20	611.1953	609.1805	$C_{28}H_{34}O_{15}$	Hesperidin*	Antioxidant, potential cardiovascular
30	17.43	517.1335	515.1188	$C_{25}H_{24}O_{12}$	Isochlorogenic acid C *	Antioxidant
31	19.78		1003.5084	$C_{48}H_{78}O_{19}$	Asiaticoside *	Wound healing, anti-inflammatory
32	19.89	595.2011	593.1859	$C_{28}H_{34}O_{14}$	Poncirin	Limited information
33	20.20		301.0356	$C_{15}H_{10}O_{7}$	Quercetin *	Antioxidant, anti-inflammatory, cardiovascular
34	20.27	285.0757	283.0610	$C_{16}H_{12}O_{5}$	Calycosin*	Estrogenic, cardiovascular
35	24.11	269.0811	267.0657	$C_{16}H_{12}O_{4}$	Formononetin *	Anti-inflammatory, estrogenic
36	22.49		299.0555	$C_{16}H_{12}O_{6}$	Rhamnocitrin	Limited information
37	23.18		263.1281	$C_{15}H_{20}O_{4}$	naphthol[1,2-b] furan-2-one	Limited information
38	26.08		871.4670	$C_{43}H_{70}O_{15}$	Astragaloside II	Immunomodulatory

* Compared with reference compounds

The chemicals contained in TQF were assessed by high-performance liquid chromatography (HPLC) spectrometry. HPLC analysis was performed using a Waters 2695 instrument (Waltham, MA, USA). The parameters were as follows: Column: Xbridge® C18, 4.6 mm×250 mm, 5 μm (Waters, USA); Mobile phase: Acetonitrile (A), water with 0.1% formic acid (B). A gradient elution system was employed:

Time (min)	% A
0-5	5-5
5-10	5-10
10-30	10-18
30-42	18
42-50	18-25
50-55	25
55-60	25-40
60-65	40

65-70	40-80
70-80	80
Detector Settings	
Wavelength	210 nm and 340 nm

As compared with standard reference compounds, eight constituents of TQF, including chlorogenic acid, cryptochlorogenic acid, calycosin 7-O-glucoside, hyperoside, hesperidin, isochlorogenic acid C, calycosin, nobiletin, were analyzed by HPLC (Fig 3 in the manuscript).
