# Supplementary material for: Renal-protective effects of Chinese medicinal herbs and compounds for diabetic kidney disease in animal models: protocol for systematic review and meta-analysis
Source: Syst Rev. 2024 Jan 12;13:23. doi: 10.1186/s13643-023-02446-4 (PMC10785383; doi:10.1186/s13643-023-02446-4)
Supplement: Supplementary file 3 — Additional file 3. Search strategy in PubMed example. [file 13643_2023_2446_MOESM3_ESM.pdf]

Here is an example of searching the animal studies of Astragali mongholici radix (Huang qi) for DKD in PubMed:

#1 "Diabetic Nephropathies" [Mesh]

#2 "Nephropathies, Diabetic"[ti/ab] OR "Nephropathy, Diabetic"[ti/ab] OR "Diabetic Nephropathy"[ti/ab] OR "Diabetic Kidney Disease"[ti/ab] OR "Diabetic Kidney Diseases"[ti/ab] OR "Kidney Disease, Diabetic"[ti/ab] OR "Kidney Diseases, Diabetic"[ti/ab] OR "Diabetic Glomerulosclerosis"[ti/ab] OR "Kimmelstiel-Wilson Syndrome"[ti/ab] OR "Kimmelstiel Wilson Syndrome"[ti/ab] OR "Syndrome, Kimmelstiel-Wilson"[ti/ab] OR "Kimmelstiel-Wilson Disease"[ti/ab] OR "Kimmelstiel WilsonDisease"[ti/ab] OR "Nodular Glomerulosclerosis"[ti/ab] OR "Glomerulosclerosis, Nodular"[ti/ab] OR "Glomerulosclerosis, Diabetic"[ti/ab] OR "Intracapillary Glomerulosclerosis"[ti/ab]

#3 #1OR#2

#4 "huang qi"[Mesh]

#5 "Astragalus mongholicus Bunge"[ti/ab] OR "astragalaus"[ti/ab] OR "astragale"[ti/ab] OR "astragali mongholici radix"[ti/ab] OR "astragali radix"[ti/ab] OR "astragali radix praeparata cum melle"[ti/ab] OR "astragalo"[ti/ab] OR "astragalus"[ti/ab] OR "astragalus mongholicus root"[ti/ab] OR "astragalus root"[ti/ab] OR "astragalus root for use in thm"[ti/ab] OR "astragálo"[ti/ab] OR "atragalus root"[ti/ab] OR "chinesischer tragant"[ti/ab] OR "hoàng ký"[ti/ab] OR "huang ci"[ti/ab] OR "huang qi"[ti/ab] OR "huang-ch"[ti/ab] OR "huangoi"[ti/ab] OR "huangq"[ti/ab] OR "huángqi"[ti/ab] OR "huángqí"[ti/ab] OR "hwanggi"[ti/ab] OR "membranous milk vetch"[ti/ab] OR "membranous milk-vetch"[ti/ab] OR "membranous milkvetch"[ti/ab] OR "meng gu huang qi"[ti/ab] OR "milkvetch"[ti/ab] OR "milkvetch root"[ti/ab] OR "mo jia huang qi"[ti/ab] OR "mongolian milk-vetch"[ti/ab] OR "mongolian milkvetch"[ti/ab] OR "neimeng huangqi"[ti/ab] OR "ogi"[ti/ab] OR "ougi"[ti/ab] OR "prepared milkvetch root"[ti/ab] OR "radix astragali"[ti/ab] OR "radix astragali praeparata cum melle"[ti/ab] OR "zhihuangqi"[ti/ab] OR "zhongfengnaomaitong"[ti/ab] OR "オウギ"[ti/ab] OR "膜荚黄耆"[ti/ab] OR "蒙古黄耆"[ti/ab] OR "黄者"[ti/ab] OR "黄耆"[ti/ab] OR "黄芪"[ti/ab] OR "黄芪"[ti/ab]

#6 #4OR#5

#7 "Animal Experimentation"[Mesh]

#8 "in vivo"[ti/ab] OR "in vivo experiment"[ti/ab] OR "in vivo experiments"[ti/ab]

#9 "rats"[ti/ab] OR "mice"[ti/ab] OR "animal models"[ti/ab]

#10 #7OR#8OR#9
